# Association between severity of illicit drug dependence and quality of life in a psychosocial care center in BRAZIL: cross-sectional study

**DOI:** 10.1186/s12955-017-0795-5

**Published:** 2017-11-17

**Authors:** Selva Rios Campêlo, Maria Alves Barbosa, Danilo Rocha Dias, Camila Cardoso Caixeta, Cláudio Rodrigues Leles, Celmo Celeno Porto

**Affiliations:** 1 Medical School, Federal University of Goiás, Rua B4, Qd 5, Lt 6, Casa 2, Setor Bueno CEP, Goiás, 74210-103 Brazil; 2 Nursing School, Federal University of Goiás, Goiás, Brazil; 3 Dentistry School, Federal University of Goiás, Goiás, Brazil

**Keywords:** Quality of life, Substance use disorders, Addiction severity, Illicit drugs, Psychiatric comorbidity

## Abstract

**Background:**

Quality of life must be one of the main purposes for the treatment of drug users, requiring a better understanding of the association between the quality of life and the severity of dependency. This study aimed to investigate the correlation between severity of substance use in various areas of human functioning and quality of life of illicit drug users in a psychosocial care center for alcohol and drugs.

**Methods:**

This cross-sectional study included 60 participants – illicit drug users – treated at a psychosocial care center for alcohol and drugs. Participants were evaluated with the short version of World Health Organization Quality of Life (WHOQOL-Bref) instrument to measure the quality of life, the 6th version of Addiction Severity Index (ASI-6) to assess the severity of dependence in several areas and the Mini International Neuropsychiatric Interview (MINI) to identify the presence of psychiatric disorders. Pearson and Spearman correlation tests and linear regression were applied to verify the association between the severity of dependence and the quality of life, and Student’s *t*-test to compare the mean quality of life between individuals with and without psychiatric comorbidities.

**Results:**

Negative correlation was found between the severity of dependence on the drugs dimensions: alcohol, psychiatric, medical, legal, family/social support and family/social problems of ASI-6, and the quality of life domains measured by the WHOQOL-Bref. The evidence was strongest in the psychiatric and medical dimensions. There was a significant difference in the quality of life mean among participants presenting or not presenting psychiatric comorbidities, for the psychological domain in anxiety disorders, and for the physical and psychological domains in mood disorders.

**Conclusion:**

The quality of life decreased as the severity of dependence increased, with different results in the various areas of the participant’s life. This result emphasizes the need for training the professional team which works in the substance use disorders area for more comprehensive diagnostic evaluations and more appropriate therapeutic interventions for each area. The associations were more evident in the medical and psychiatric fields, indicating the need for greater attention to be paid in relation to medical and psychiatric comorbidities.

**Electronic supplementary material:**

The online version of this article (10.1186/s12955-017-0795-5) contains supplementary material, which is available to authorized users.

## Background

The worldwide prevalence of illicit drug use was estimated at 5.2% and problematic consumption by 0.6% in 2013 [1]. While cannabis use has been increasing since 2009, the use of opiates has stabilized at high levels, and the use of amphetamines and cocaine has decreased in general. However, in South America cocaine use has not decreased; instead, the annual prevalence increased from 0.7% in 2010 to 1.2% in 2012, which corresponds to three times the estimated mean level of global consumption [1]. Brazil, among the countries of South America, has the largest cocaine market [1], which may contribute to increased risk for the population to develop problematic use or dependence of this substance or its derived, such as crack.

For the World Health Organization (WHO) the abuse of illicit drugs is a global problem that requires health programs to minimize the risk of death and related infectious diseases such as human immunodeficiency virus (HIV) and hepatitis B and C. Treatments for substance use disorders are carried out by Brazilian public health in community-based psychosocial care centers that aim to reduce the harm [2]. In other words, they do not focus only on the amount and frequency of drug use, but also on improving the users’ quality of life, seeking for solutions to the consequences of misuse.

Since health is no longer considered just the absence of disease (according to WHO), but as one’s complete physical, psychological, and social well-being, quality of life has been considered a health indicator, for both assessments and treatment outcomes [3]. In the field of public policies, quality of life allows identifying the population’s health needs, to elect the priorities of assistance and to compare the results of the different treatments performed [4]. The development of the quality of life’s instruments and their use as a comprehensive parameter of health assessment [3, 5] allowed an evaluation, in the field of substance use disorders, focused not only on the amount and frequency of drug use or on biological factors. Instead, these instruments assess the extent that the disorder can cause in people’s lives in various domains of human functioning [6] and in their self-perception on some aspects related to drug abuse, such as traumatic symptoms, living conditions and social support [7].

Quality of life has been used with distinct goals in the drug dependency area: 1) As a variable of therapeutic results [8–11]; 2) To describe groups of people with substance use disorders, and to compare them with the general population, with people presenting other types of disorders, and even among subpopulations of drug users [12–17]; 3) As reference instrument in the validation process of new instruments of quality of life [18–20]; and 4) To test its association with other variables related to abuse or dependence [21–28].

The severity of dependence evaluated in different aspects, such as medical, psychiatric, legal, family/social and employment/finances, is an important variable to be studied in relation to user’s quality of life [29, 30]. It is differentiated in each aspect for each individual person and is not exclusively related to the pattern of substance use [31–34]. Knowledge of the relationship between severity of dependence and quality of life promotes the raising of evaluative and therapeutic possibilities in treatment for abuse and substance dependence.

Understanding the real needs of drug users, and the inclusion of psychosocial parameters can help for building more consistent therapeutic projects for each person. Health institutions and public policies may use all these information for setting goals and defining priorities of scheduling and of treatments.

Thus, the aim of this study was to investigate how the severity of illicit drug dependence in various areas of human functioning is related to the quality of life as perceived by users.

## Methods

This cross-sectional study was carried out with illicit drug users in outpatient treatment in a psychosocial care center for alcohol and drugs (CAPSad) in Goiânia, Goiás, Brazil, from June 2015 to February 2016. This service is offered by the Single System Health (SUS) in Brazil for adult people with substance use disorders. The service is carried out to reduce harm and aims the psychosocial recovery of users in addition to medical treatment. Up to 2013, it was the only referral service in Goiânia for the adult population and, therefore, has users from all parts of the city.

### Sample

The sampling process was non-probabilistic. All individuals with illicit drug problems being followed at the unit searched from June 2015 to February 2016 were included, since they accepted to participate in the study and who fulfilled the inclusion and exclusion criteria of the study. Around 500 users are monitored regularly in the unit and, from these, 220 present illicit drugs problems.

The invitation to collaborate with the study occurred during therapeutic group sessions. Since the treatment occurs according to an outpatient care model, the groups had a variable number of participants, and their frequency was inconstant. All the illicit drug users present in the groups were informed about the objectives, procedures and ethical aspects of the research. Those who agreed to participate, who were 18 years or older, independent of the use of alcohol, were included in the sample, and the interviews were scheduled for data collection. Therefore, only those who had the scheduled interviews were submitted to evaluation by the research instruments.

The users who did not present cognitive condition at the time of the interview, due to the presence of severe neurological or psychiatric symptoms, were excluded. This condition was identified by means of clinical diagnosis performed by a psychologist (the main researcher - SRC).

### Procedures

The data collection was carried out by the main researcher (SRC) and trained assistant researchers who were supervised and had their procedures calibrated before the application of the instruments. This training was performed according to instructions from the team of the Center for Research on Alcohol and Drugs, from the psychiatry department of the Federal University of Rio Grande do Sul, Brazil, which carried out the validation of Brazilian version of the Addiction Severity Index - ASI-6 [35].

The following instruments were used: World Health Organization Quality of Life - Bref (WHOQOL-Bref) for the quality of life assessment; Addiction Severity Index (ASI-6) to assess the severity of dependency and Mini International Neuropsychiatric Interview – version Core (MINI Core) to explore the existence of psychiatric comorbidities. The WHOQOL-Bref was self-applied and assisted, the other two instruments were applied by the researchers.

The WHOQOL-Bref is the abbreviated form of the WHOQOL-100 instrument developed by WHO to evaluate the quality of life. It was validated in Brazilian version by Fleck et al. [36] and presented satisfactory characteristics of internal consistency, discriminant validity, criterion validity, concurrent validity and test-retest reliability. The questionnaire contains 26 questions, of which two are general questions about overall quality of life and general perception of health, and the remaining 24 represent each of the 24 facets of the original instrument, subdividing into four domains: 1) Physical (pain, medication, energy, mobility, sleep, work); 2) Psychological (positive feelings, spirituality, thought, body, esteem, negative feelings); 3) Social relations (relationships, sex, support); and 4) Environment (security, finance, information, leisure, home, services, transportation). Responses follow a Likert scale from 1 to 5. The scores are calculated separately in each domain and transformed on a scale from 0 to 100. The better the score, the better the quality of life [36].

The ASI is a semi-structured multidimensional interview that aims to measure the severity of substance dependence. The sixth version of the instrument - ASI-6 - was validated in Brazil in a multicenter study, coordinated by Kessler and Pechansky [30, 35], sponsored by the National Secretariat for Policy on Drugs (SENAD), demonstrating good reliability and validity for the Brazilian culture. The scale evaluates seven dimensions of life functioning: medical, employment/finances, legal, psychiatric, alcohol, other drugs and family/social. In each of these dimensions, there are 3 types of questions to evaluate symptoms and problems: the whole life of the individual, in the previous six months, and the recent problems in the last 30 days. One score is calculated for each dimension, except for the family/social area, which is subdivided into 3 scores: family/social problems, family/social support and family/child. The result varies from 0 to 100, and the higher the score, the greater the severity of the dependency [37].

The MINI is a standardized diagnostic interview, compatible with the diagnostic criteria for psychiatric disorders according to the International Classification of Diseases (ICD-10) and the Diagnostic and Statistical Manual of Mental Disorders (DSM-IV), which allows the reduction of variability in diagnoses performed, favoring comparisons in epidemiological studies [38]. It presented good validity and reliability in studies conducted in Europe and the United States [39]. The translation into Portuguese was carried out by a Brazilian research who was part of the original development group of the instrument [38, 39]. The interview features sixteen modules that explore DSM-IV specific disorders. Fifteen modules explore axis I specific disorders - anxiety, mood, eating disorders, alcohol and other substances dependence/abuse, psychotic disorder. A module explores the antisocial personality of axis II. For the present study were excluded the modules of eating disorders because it was not the interest of this research, and those of dependence/abuse of alcohol and substances because they were already included in ASI-6.

### Statistical analysis and results

Descriptive and inferential statistics were carried out using SPSS, Statistical Program of the Social Sciences, version 20.0. The association between quality of life and severity of dependence was analyzed using Pearson and Spearman correlation tests and multiple linear regression. The Student’s *t*-test was used to compare the quality of life mean between individuals who had or not psychiatric comorbidities.

### Ethical aspects

This project was approved by the Institutional Ethics Committee of the Federal University of Goiás (Reference number 927256) (Additional files 1 and 2). All participants agreed to participate by signing an Informed Consent. They were aware that participation was not mandatory and that they could withdraw their consent without any loss of continuity of treatment at the institution.

## Results

A total of 72 illicit drug users being treated at the unit agreed to participate after receiving the invitation. Of these, two users were excluded because they presented low cognitive conditions due to psychiatric symptoms and ten did not attend the scheduled interviews, resulting in 60 participants, who constituted the study sample.

The demographic data are shown in Table 1. The participants were predominantly male, unemployed, living alone, and most of them had completed a high school education.

**Table 1:** Socio-demographic data of illicit drug users, CAPSad - Brazil, 2016

Variables	Mean (SD)	N (%)
Age	38.0 (9.9)	
Sex		
Male		55 (91.7)
Female		5 (8.3)
Employment^a^		
Unemployed		42 (70.0)
Employed		18 (30.0)
Partner situation		
Without partner		44 (73.3)
With partner		16 (26.7)
School level		
Elementary level incomplete		15 (25.0)
Elementary level complete		15 (25.0)
High school complete		28 (46.7)
Graduation complete		2 (3.3)

^a^Employed did not include irregular employment

Details of the types of substances and patterns of use are described in Table 2. It was observed that of the 60 illicit drug users surveyed, 98.3% also had used alcohol at least once, of which 45.8% used alcohol in the last 30 days. Regarding illicit drug use, marijuana was the more frequently used at least once, the most used in the previous 30 days, with the longer time of regular use, and with the lowest age of first use. After marijuana, crack and inhaled cocaine presented similar frequency for use at least once and for regular use. The use of crack began the latest compared to all the other substances but had the second most frequency of use within the previous 30 days.

**Table 2:** Usage patterns of psychoactive substances by illicit drug users, CAPSad - Brasil, 2016

	Used at least once	Used in the previous 30 days^a^	Years of regular use	Age at 1st use
Substances	N (%)	N (%)	Mean (SD)	Mean (SD)
Alcohol	59 (98.3)	27 (45.8)	12.4 ± 10.9	13.7 ± 4.5
Marijuana	59 (98.3)	25 (42.4)	11.1 ± 10.0	15.7 ± 5.3
Inhaled cocaine	52 (86.7)	12 (23.1)	5.6 ± 7.1	20.4 ± 7.3
Crack^b^	51 (85.0)	17 (33.3)	5.7 ± 6.0	25.3 ± 9.8
Inhalants	45 (75.0)	2 (4.4)	2.0 ± 3.7	16.4 ± 6.2
Hallucinogens	26 (43.3)	1 (3.8)	1.0 ± 2.2	22.3 ± 7.9
Stimulants	16 (26.70)	2 (12.5)	1.6 ± 3.8	24.1 ± 6.7
Heroin	5 (8.3)	0 (0.0)	0.6 ± 0.8	20.2 ± 6.0
Other opioid	2 (3.3)	0 (0.0)	5.0 ± 0.0	21.5 ± 7.7

^a^Percentage calculated in relation to users who used each substance at least once

^b^Crack represents the crack/merla/oxy group

**Fig. 1:**
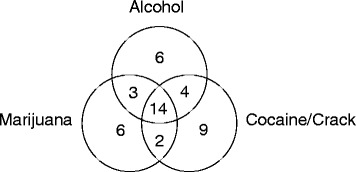
Frequency of concomitant use of two or more substances

The concomitant use of two or more substances in the past 30 days, including alcohol, was represented in Fig. 1. Regarding the participant’s perception about the drugs considered as a cause of problems and reason for seeking treatment, independent of use in the previous 30 days, forty-two participants indicated two substances and ten indicated three, reflecting the high use frequency of multiple drugs.

The descriptive results of WHOQOL-Bref domains and ASI-6 dimensions are described in Table 3, and the association between them is described in Table 4. In general, the severity of dependence was correlated with quality of life, in an inversely proportional way. The only dimensions of ASI that presented no correlations with WHOQOL-Bref domains were family/child and employment/finances. While alcohol use correlated with the physical and psychological domains of quality of life, the drug use was correlated only with the physical domain. Psychiatric and medical dimensions of ASI seems to affect all the domains of quality of life, except the medical dimension for environment domain.

**Table 3:** Descriptives of WHOQOL-Bref and ASI of illicit drug users, CAPSad - Brazil, 2016

WHOQOL	Mean (SD)	ASI	Mean (SD)
Physical	56.2 (17.8)	Drugs	49.4 (6.8)
Psychological	54.3 (20.6)	Child Problems	51.8 (7.3)
Social	47.8 (25.6)	Alcohol	50.1 (9.5)
Environmental	49.2 (16.3)	Psychiatric	49.9 (7.8)
		Medical	48.2 (8.6)
		Legal	48.7 (5.1)
		Employment/finances	41.6 (11.3)
		Family/Social Support	49.7 (10.4)
		Family/Social Problem	52.5 (8.8)

**Table 4:** Correlations between ASI-6 and WHOQOL-Bref of illicit drug users, CAPSad - Brazil, 2016

	WHOQOL domains			
ASI-6 dimensions	Physical	Psychology	Social	Environmental
Drug	−0.420**	−0.189	−0.228	−0.133
Family/Child	0.430	0.247	0.131	0.244
Alcohol	−0.363**	−0.369**	−0.075	−0.203
Psychiatric	−0.497**	−0.510**	−0.327*	−0.301*
Medical	−0.314*	−0.343**	−0.409**	−0.187
Legal	−0.125	−0.280*	−0.191	−0.269*
Employment/finances	−0.204	−0.194	−0.113	−0.010
Family Social Support	−0.166	−0.153	−0.076	−0.291*
Family Social Problem	−0.142	−0.174	−0.263*	−0.019

* *P* < 0.05 - significant coefficients of Pearson or Spearman (legal)

** *P* < 0.01 - highly significant coefficients of Pearson

The variables that were significantly correlated with each specific domain of quality of life (*p* < 0,05, Table 4) were included in the multiple linear regression analysis, besides socio-demographic data (employment, partner situation and school level). The results suggested that the main predictors of physical domain of quality of life (r^2^ = 0,38) were the psychiatric dimension (β = −0,34, *p* = 0,004), use of drugs (β = −0,36, *p* = 0,003), and partner situation (β = −0,24, *p* = 0,035). The psychological domain was main affected by the psychiatric dimension of ASI (β = −0,51, *p* = 0,000, r^2^ = 0,26). The social domain was mainly affected by medical dimension (β = −0,41, *p* = 0,001, r^2^ = 0,17) and the environmental domain by the partner situation (β = −0,4, *p* = 0,001) and school level (β = 0,3, *p* = 0,011) (r^2^ = 0,25).

The frequency of psychiatric comorbidities was high in this sample, especially anxiety and mood disorders (Table 5). There was a significant difference in the quality of life means in the psychological domain between the participants who did or did not present anxiety disorders, and in the physical and psychological domains between those who did or did not present mood disorders.

**Table 5:** Comparison of the means of WHOQOL-Bref domains for the presence of comorbidities of illicit drug users, CAPSad, Goiás, Brazil, 2016

		WHOQOL Domains			
		Physical	Psychology	Social	Environmental
Comorbidities	N (%)	Mean (SD)	Mean (SD)	Mean (SD)	Mean (SD)
Anxiety	45 (75.0)				
Yes		53.53 (17.12)	50.81 (18.71)	45.24 (24.29)	47.96 (14.11)
No		62.81 (18.26)	62.55 (23.11)	53.70 (28.33)	52.26 (20.61)
*P*		0.069	0.031*	0.107	0.205
Mood	42 (70.0)				
Yes		53.06 (16.81)	50.24 (18.84)	46.30 (23.67)	49.28 (16.92)
No		66.33 (17.70)	66.61 (21.59)	52.22 (31.25)	49.17 (14.68)
*P*		0.014*	0.004*	0.211	0.746
Psychotic	35 (58.3)				
Yes		55.71 (18.54)	54.90 (19.97)	49.05 (24.90)	50.23 (15.13)
No		56.92 (17.05)	53.53 (21.97)	46.00 (27.02)	47.87 (17.97)
*P*		0.801	0.856	0.866	0.746
Antisocial	25 (41.7)				
Yes		56.29 (18.33)	56.83 (19.94)	49.00 (27.57)	50.00 (17.09)
No		56.14 (17.69)	52.5 (21.25)	46.90 (24.51)	48.71 (15.89)
*P*		0.976	0.463	0.915	0.891

*P significant for difference of the average between presence and absence of comorbidity, by Student’s *t*-test

## Discussion

The findings of this research revealed that quality of life decreases as the severity of dependence increases, corroborating with results of other studies that associated a poor quality of life with the severity of dependence [26, 30, 40, 41]. The evidence of this negative correlation between the quality of life domains and the dependence severity was strongest in psychiatric and medical dimensions and weaker in family/social support, family/social problems, and legal dimensions. These results were consistent with previous studies that highlighted the negative correlation between the severity of psychiatric and medical disorders and quality of life [22, 26, 27].

The employment/finances and child problems dimensions of ASI-6 were not associated with any WHOQOL-Bref domains in this study. Maybe this result can be explained by the weaker psychometric properties of these dimensions in comparison with the others, as discussed by some validation studies [29, 42].

Drug users usually seek treatment when they experience serious consequences as result of their dependence. In other words, the quality of life is worse in people with abuse and dependence on drugs seeking treatment than in people without these disorders or other chronic conditions [41, 43, 44]. This affirmation can be confirmed when comparing the mean quality of life found in our sample with the quality of life of the population assessed by Fröhlich et al. (2010) [45] in a Family Health Strategy service in Brazil, which is a preventive care for the general population. The authors investigated if the quality of life could be related to psychotropic drugs prescription. Individuals who did not receive psychotropic drugs prescription (*n* = 274) presented quality of life mean scores around 20% higher than our sample, for each domain.

The decrease in quality of life of drug users cannot be explained only by the frequency or amount of substance use [6, 33, 40, 46], but by negative effects in several areas of their lives, with different levels of severity [47]. Investigating the severity of dependence and its relation to the quality of life of the drug user makes it possible to understand the diagnostic and therapeutic possibilities by health professionals [22, 40, 48]. This allows the identification of areas with greater severity and negative impact as perceived by the users themselves, to be prioritized in treatment. Thus, health professionals should be trained and encouraged to use the quality of life instruments in their work routine so that, over time, they can understand in which domains the treatment has been effective and in which needs to be better targeted. In addition, these instruments can be used as service evaluation tools since they make it possible to measure how much the treatment offered has, in fact, impacted the quality of life of the users served. Furthermore, these instruments provide quantitative data of subjective aspects of treatment outcomes. They make possible, from researches projects, to compare different treatments, different groups and different services, contributing to the evidence-based practice, by means of scientific knowledge.

Our results showed the psychiatric dimension of the ASI-6 as a predictor of physical and psychological domains of quality of life, which highlight the importance of a greater attention to the psychiatric comorbidities in treatment for drug abuse and dependence. Chronic drug users have a high prevalence of psychiatric disorders, which can be independent of drug use or its consequence [49], and may compromise their clinical and social evolution [50]. Thus, diagnostic, therapeutic, and prognostic criteria of comorbidities must be highlighted, which has been done in studies mainly in Europe and the United States [51]. Health professionals who deal with the issue of abuse and drug addiction, such as the professionals of CAPSad, must be prepared to assess and intervene competently with comorbid disorders, which are common in the substance abuse area. Their training should be not only in theoretical knowledge but also in the development of skills and attitudes [52], that can promote both the health and changes in the behavior and lifestyle of the drug user.

An epidemiological study of the American population, the Epidemiologic Catchment Area (ECA), found a prevalence of 53% of psychiatric comorbidity among those who abuse or are dependent on alcohol and other drugs, and the authors suppose that in mental health treatment environments, this rate should be higher [53]. Mood disorders and anxiety disorders are the most frequent among drug users [50, 54]. A study of the National Epidemiological Survey on Alcohol and Related Conditions (NESARC) showed that among users seeking treatment, 40.69% had a mood disorder and 33.38% had an anxiety disorder [55]. In Brazil, there are few studies investigating the prevalence of psychiatric comorbidities among drug users. A study in CAPSad found a high prevalence of comorbidities, and the most observed diagnoses were major depressive episodes with 69.9% and generalized anxiety with 63.10% [50]. Other studies have shown that anxiety disorders [14, 27] and mood disorders [24, 26] are very prevalent and have a negative effect on abusers/dependents quality of life. The evaluation by MINI in this study agrees with these previous studies identifying a high prevalence of mood and anxiety comorbidities.

Previous studies indicate that most of the crack users are male, young, without a partner, have a low socio-economic status, low level of education, and are unemployed [56–58]. Marijuana users seeking treatment in Brazil tend to be male, with an average age of 32, with no partner, and have some degree of education and occupation [59]. Recent studies in CAPSad have shown a different profile of illicit drugs users – for example, a higher average age [26], higher education [60], or higher socio-economic status [24, 60] – similar to our findings.

The high prevalence of marijuana found in this research is in line with the fact that it is the most widely used illicit drug worldwide [1]. Its high use in the previous 30 days indicates a high rate of continuity of use after experimentation. The earlier someone begins using marijuana and the longer use, the more harmful the consequences can be [61].

This study observed that, after marijuana, inhaled cocaine and crack cocaine were the most commonly used illicit drugs, confirming the high prevalence of these substances in Brazil [1]. A multicenter study revealed an increase in the prevalence of crack use in Brazil and highlighted the severity of psychosocial problems and psychiatric symptoms in crack users [62]. Other study showed that 78.9% of crack users in treatment at CAPSad used another substance before crack [60].

Changes in the profile of populations with substance abuse reveal an increased use of multiple drugs rather than the use of only one substance [48, 57], which may lead to a greater severity of dependence [48]. This new reality was also observed in our sample. The knowledge about which is the substance first experimented, the longest used and the most recently used, besides which of them are used concomitantly, may add information about the drug users’ environment and may guide public policies to prevent the use of these substances. A research suggested that the sequence of drugs’ first use may be more related to external factors such as group pressure and trafficking influence, rather than user preference [56]. The low percentage of inhalants, as well as stimulants and hallucinogens, may be justified by external factors, such as the ease of obtaining other drugs, such as crack cocaine, which is easy to market in Brazil [1, 56].

Even with the limitations of sample size and its heterogeneity of substance use at the moment of the interview, this study makes important contributions, showing the need to know the different aspects that should be highlighted in the treatment of substance use disorders. Treatment that does not prioritize user needs, which can be revealed in subjective assessments of quality of life, commonly has high dropout rates [37]. The fact that the quantity and frequency of substance use are not the most salient factors to determine the quality of life in this population implies the need for treatment and services that focus on areas other than sobriety or reduction of use [7, 46], such as user recovery. The recovery may include physical and mental health, social functioning, safe environment, comfort, and availability of resources.

The results of this study emphasize the importance of further studies on the presence of psychiatric disorders in users who receive treatment for substance abuse/dependence, and on how the presence of psychiatric comorbidities can be related to the quality of life of these users. Another aspect to be highlighted is the perception that there is a need for capacity-building for professionals from different areas that are involved in the treatment so that there can be an improvement in both diagnostic evaluation and therapeutic interventions, reinforcing the integrality in the care of CAPSad users.

## Conclusion

In conclusion, this study revealed the inverse correlation between the severity of dependence on illicit drugs and the quality of life of users. These findings highlight the importance of assessing the quality of life of illicit drug users in treatment, and of understanding how it may be related to the various aspects of their lives. This understanding can facilitate an assessment of the problem, the development of approach strategies and therapeutic intervention, and more appropriate public policies for substance use disorders.

## Additional files

Additional file 1: Approval Document from the Ethics Committee of the Federal University of Goiás, Brazil. (PDF 36 kb)

Additional file 2: Research consent by the institution “Psychosocial care center for alcohol and drugs” (CAPSad). (PDF 486 kb)

## Abbreviations

ASI: Addiction Severity Index
CAPSad: Psychosocial Care Center for alcohol and drugs
DSM-IV: Diagnostic and Statistical Manual of Mental Disorders
ECA: Epidemiologic Catchment Area
HIV: Human Immunodeficiency Virus
ICD-10: International Classification of Diseases
MINI: Mini International Neuropsychiatric Interview
NESARC: National Epidemiological Survey on Alcohol and Related Conditions
SENAD: National Secretariat for Policy on Drugs
SPSS: Statistical Program of the Social Sciences
SUS: Single System Health
WHO: World Health Organization
WHOQOL: World Health Organization Quality of Life
