# Supplementary material for: Association between severity of illicit drug dependence and quality of life in a psychosocial care center in BRAZIL: cross-sectional study
Source: Health Qual Life Outcomes. 2017 Nov 17;15:223. doi: 10.1186/s12955-017-0795-5 (PMC5693571; doi:10.1186/s12955-017-0795-5)
Supplement: Supplementary file 1 — Approval Document from the Ethics Committee of the Federal University of Goiás, Brazil. (PDF 36 kb) [file 12955_2017_795_MOESM1_ESM.pdf]

PARECER CONSUBSTANCIADO DO CEP

DADOS DO PROJETO DE PESQUISA

Título da Pesquisa: QUALIDADE DE VIDA DOS USUÁRIOS DE DROGAS ILÍCITAS ATENDIDOS NOS CENTROS DE ATENÇÃO PSICOSSOCIAL ÁLCOOL E DROGAS DE GOIÂNIA

Pesquisador: Selva Rios Campêlo

Área Temática:

Versão: 3

CAAE: 37419814.5.0000.5078

Instituição Proponente: Hospital das Clínicas Universidade Federal de Goiás - GO

Patrocinador Principal: Financiamento Próprio

DADOS DO PARECER

Número do Parecer: 927.256

Data da Relatoria: 17/12/2014

Apresentação do Projeto:

Projeto investiga a Qualidade de Vida(QV)dos usuários de drogas ilícitas no início do atendimento e em fases posteriores de acompamento nos CAPS de Goiânia. Sendo um estudo transversal,descritivo e analítico que utiliza amostras constituídas por usuários que deram entrada no CAPS no período de 2 meses após início da coleta de dados.Avaliados em 3 momentos diferentes de inserção e a coleta de dados será através de questionário de QV WHOQOL-Bref e questionário sóciodemográfico,totalizando amostragem de 100 usuários.

O consumo de drogas é um fator significativo de risco para a morbidade e mortalidade no Brasil e em todo mundo. Segundo a Organização Mundial de Saúde (OMS) seu impacto deve ser enfrentado por políticas de saúde que contemplem também as questões sociais envolvidas (WHO, 2004).A política de atenção integral a usuários de álcool e drogas do Ministério de Saúde no Brasil está de acordo com a perspectiva apresentada pela OMS de se considerar os determinantes sociais da saúde, tais como condições socioeconômicas, culturais e ambientais gerais. Propõe que o atendimento das necessidades dos usuários de álcool e drogas atendidos pelo sistema de Saúde Pública deve ser realizado de forma ampla, englobando ações de prevenção, promoção, tratamento e reabilitação (BRASIL 2003). Partindo desta concepção mais ampla do processo saúde-

Endereço: 1ª Avenida s/nº - Unidade de Pesquisa Clinica
Bairro: St. Leste Universitario **CEP:** 74.605-020
UF: GO **Município:** GOIANIA
Telefone: (62)3269-8338 **Fax:** (62)3269-8426 **E-mail:** cephcufg@yahoo.com.br

doença na qual se considera os determinantes sociais da saúde afirma-se que a melhora da Qualidade de Vida deve estar entre os principais objetivos do tratamento para abuso e dependência de drogas (BRASIL, 2003; LAUDET, 2011; TIFFANY et al 2012). Verifica-se assim a importância que pode assumir a Qualidade de Vida na avaliação e monitoramento de tratamentos e de políticas públicas para a área de transtornos relacionados ao uso de substâncias psicoativas. JUSTIFICATIVA DO ESTUDO A despeito da importância que pode assumir a Qualidade de Vida (QV) na área de
transtornos relacionados ao uso de substâncias constata-se que existem poucos estudos envolvendo QV no tratamento das pessoas com abuso e dependência de drogas (TIFANY et al, 2012). Para avaliação da QV existem diferentes tipos de instrumentos que se classificam em três categorias relacionados ao desfecho que se busca avaliar. A primeira categoria mede a QV geral. Está relacionada a um modelo de satisfação e bem estar e busca a avaliação de objetivos amplos de satisfação da pessoa com a vida e bem estar social. A segunda e terceira categorias estão relacionadas a um modelo funcionalista no qual se considera principalmente o status de saúde e funcional da pessoa, medem, por isso, a Qualidade de Vida Relacionada à Saúde (QVRS). A diferença entre estas duas últimas categorias está que enquanto a segunda categoria mede de forma genérica a QVRS, ou seja, para qualquer doença a terceira categoria a mede de forma específica a uma doença (LIMA, 2002; FLECK, 2008). A área do abuso e dependência de drogas iniciou tardiamente a utilização da QV como forma de avaliação de resultado de tratamento em relação a outras áreas de doenças crônicas (LAUDET, 2011), o que justifica o fato de existirem poucos instrumentos de QV específicos para esta área já validados. A maioria dos estudos com foco na QV em pesquisas na área de abuso e dependência de drogas utilizam instrumentos genéricos de QVRS (LAUDET, 2011).No Brasil ainda não existe nenhum instrumento de QV específico para transtornos por uso de substâncias validado e constata-se também um
número muito pequeno de estudos que investigam a interação entre dependência de drogas e a QV, o que pode ser devido à disponibilidade limitada de instrumentos validados (CARLOS ZUBARAN et al, 2009). Apesar de que a maioria dos estudos utilizarem medidas de QVRS genéricas a QV global ou geral deveria ser mais utilizada do que instrumentos de QVRS genéricos devido ao fato de relacionar-se mais diretamente com metas de recuperação dos usuários de drogas, pois além de estarem livres de sintomas, indivíduos em recuperação expressam preocupações em múltiplas áreas de funcionamento avaliadas no WHOQOL, considerado um instrumento padrão ouro para a avaliação da QV geral (LAUDET, 2011). Diante disso, este projeto se mostra relevante para a área da Ciências da saúde, pois procura

compreender a utilização da Qualidade de Vida como forma de avaliação para o tratamento do abuso e dependência de substâncias psicoativas, proporcionado pelo sistema público de saúde, o que pode reforçar reflexões tanto dos profissionais quanto dos gestores da saúde.

Metodologia Proposta:
Trata-se de um estudo de coorte que passará pelo crivo do Comitê de Ética Institucional e da Universidade Federal de Goiás. O desenvolvimento deste projeto será nos três CAPS ad para população maiores de 18 anos existentes em Goiânia, O CAPS CASA localiza-se no distrito sul de saúde, o CAPS Noroeste no distrito noroeste e o CAPS Negrão de Lima no distrito campinas/centro. Estes CAPS são administrados pela secretaria municipal de saúde de Goiânia e atendem usuários de álcool e outras drogas acima de 18 anos e seus familiares pelo Sistema Único de Saúde (SUS). A abordagem desenvolvida é psicossocial, e a equipe é formada por diferentes profissionais, contando com médicos generalistas e psiquiatras, enfermeiros, farmacêuticos, psicólogos, assistentes sociais, arteterapeutas, terapeutas ocupacionais, profissionais da educação física e musicoterapeutas. A amostragem do estudo será constituída por uma amostra de conveniência incluindo todos os
indivíduos que cumprirem os critérios de inclusão e exclusão e derem entrada em um dos três CAPS ad do presente estudo nos 2 meses iniciais da coleta de dados. Os Critérios de inclusão são usuários acolhidos na instituição do sexo masculino e com idade de 18 anos ou acima que apresentem problemas ou dependência de drogas ilícitas, com possibilidade do uso de álcool associado desde que não seja a droga de preferência. Critérios de
exclusão são usuários que não concordarem em participar da pesquisa, ou mesmo que não apresentarem condições cognitivas de compreensão do TCLE. Usuários que não participarem de pelo menos sete encontros na avaliação em dois meses de acompanhamento e pelo menos doze encontros em quatro meses de acompanhamento serão excluídos do estudo longitudinal. Os encontros tratam-se das participações dos usuários na instituição, podendo ser encontros grupais ou individuais com qualquer profissional técnico em saúde. Os instrumentos para a coleta de dados serão: questionário de Qualidade de Vida WHOQOL-Bref (ANEXO) validado no Brasil pelo pesquisador Dr. Marcelo Pio de Almeida Fleck (2000), e
questionário sociodemográfico elaborado com os seguintes itens: idade, estado civil, nível de escolaridade, situação de emprego e renda, modos/frequência/ preferência das drogas consumidas (ANEXO). O WHOQOL-Bref é um instrumento multidimensional desenvolvido pela OMS, constituídode 26 perguntas, das quais 2 são sobre a Qualidade de Vida geral e as restantes

se subdividem em 4 domínios: físico, psicológico, relações sociais
e meio ambiente. As respostas seguem uma escala de Likert de 1 a 5, quanto maior a pontuação melhor a Qualidade de Vida. A amostra será avaliada no momento após o acolhimento e em dois momentos posteriores ao atendimento, com dois meses e com quatro meses. Todos os usuários que forem acolhidos serão avaliados após a aprovação do Comitê de Ética. Os sujeitos que responderem os critérios de inclusão e exclusão responderão inicialmente o questionário sociodemográfico e em seguida o questionário de Qualidade de Vida WHOQOL-Bref em três momentos diferentes. Para compreensão dos dados colhidos será realizada estatística descritiva e analítica utilizando-se o programa estatístico
SPSS (Statistical Packagefor Social Sciences, versão 15.0). A estatística descritiva será usada para a ordenação dos dados sociodemográficos em tabelas ou gráficos. Para a leitura e análise dos dados colhidos do WHOQOL-Bref será usada a estatística analítica. Para analisar as alterações nos escores das dimensões física, psicológica, social e ambiental da QV dos usuários de drogas ilícitas com 2 meses e 4 meses de acompanhamento nos CAPS ad de Goiânia será utilizado o teste de comparação pareada. Para analisar a influência de variáveis clínicas e sociodemográficas nas alterações das dimensões física, psicológica, social e ambiental de QV será utilizado análise fatorial multivariada.
Critério de Inclusão:
Os Critérios de inclusão são usuários acolhidos na instituição do sexo masculino e com idade de 18 anos ou acima que apresentem problemas ou
dependência de drogas ilícitas, com possibilidade do uso de álcool associado desde que não seja a droga de preferência. Usuários que não
participarem de pelo menos sete encontros na avaliação em dois meses de acompanhamento e pelo menos doze encontros em quatro meses de
acompanhamento serão excluídos do estudo longitudinal. Os encontros tratam-se das participações dos usuários na instituição, podendo ser
encontros grupais ou individuais com qualquer profissional técnico em saúde.
Critério de Exclusão:
Critérios de exclusão são usuários que não concordarem em participar da pesquisa, ou mesmo que não apresentarem condições cognitivas de
compreensão do TCLE.

Objetivo da Pesquisa:

Objetivo Primário:

Investigar a Qualidade de Vida (QV) dos usuários de drogas ilícitas no início do atendimento e em fases posteriores de acompanhamento nos Centros de Atenção Psicossocial álcool e drogas (CAPS ad) de Goiânia.
Objetivo Secundário:
Avaliar as dimensões física, psicológica, social e ambiental da QV dos usuários de drogas ilícitas que iniciam atendimento e com 2 meses e 4 meses de acompanhamento nos CAPS ad de Goiânia.Analisar as alterações nos escores das dimensões física, psicológica, social e ambiental da QV dos usuários de drogas ilícitas com 2 meses e 4 meses de acompanhamento nos CAPS ad de Goiânia. Analisar a influência de variáveis clínicas e sociodemográficas dos usuários nas alterações das dimensões física, psicológica, social e ambiental de QV.

Avaliação dos Riscos e Benefícios:
Conforme o texto do TCLE , foi explicado ao sujeito participante os riscos e benefícios da pesquisa , após solicitação de adequação do TCLE:
Existe o risco do participante sentir-se constrangido com a pesquisa. O participante não será obrigado a participar caso isto ocorra. No entanto a pesquisa será conduzida para que não haja riscos, prejuízos ou desconfortos provocados pela participação. Não haverá nenhum tipo de pagamento ou gratificação financeira pela sua participação. Os benefícios decorrentes da participação da pesquisa serão as possibilidades de melhoria nos serviços públicos de atendimento aos usuários de álcool e drogas advindos dos resultados dos estudos. O período de participação será somente o da administração dos 2 questionários citados acima, o que deverá ter duração média de 30 minutos.
O conteúdo avaliado é sigiloso e os dados coletados serão armazenados no CAPS ad CASA para possíveis estudos futuros, que em caso de utilização, o projeto de pesquisa será submetido para análise de um Comitê de Ética em Pesquisa. Você tem a garantia de aceitação ou não do presente estudo, bem como o de retirar o consentimento, sem qualquer prejuízo da continuidade do acompanhamento e tratamento usual.

Comentários e Considerações sobre a Pesquisa:
A pesquisa apresenta importância podendo apontar futuramente sugestões para melhoria na qualidade do atendimento ao usuário do CAPS.

Endereço: 1ª Avenida s/nº - Unidade de Pesquisa Clinica
Bairro: St. Leste Universitario **CEP:** 74.605-020
UF: GO **Município:** GOIANIA
Telefone: (62)3269-8338 **Fax:** (62)3269-8426 **E-mail:** cephcufg@yahoo.com.br

Considerações sobre os Termos de apresentação obrigatória:

A pesquisadora atendeu as alteraçãoes solicitadas no TCLE, explicando sobre os riscos da pesquisa.

Recomendações:

Alterar no resumo do projeto quanto aos riscos e benefícios,conforme citações do TCLE

Conclusões ou Pendências e Lista de Inadequações:

PENDENCIAS ATENDIDAS

Situação do Parecer:

Aprovado

Necessita Apreciação da CONEP:

Não

Considerações Finais a critério do CEP:

Diante do exposto, a Comissão de Ética em Pesquisa do Hospital das Clínicas/UFG - CEP/HC/UFG, de acordo com as atribuições definidas na Resolução CNS 466/12, manifesta-se pela aprovação do projeto de pesquisa proposto.

Após início, o pesquisador responsável deverá encaminhar ao CEP/HC/UFG, via Plataforma Brasil, relatórios trimestrais/semestrais do andamento da pesquisa, encerramento, conclusões e publicações. O CEP/HC/UFG pode, a qualquer momento, fazer escolha aleatória de estudo em desenvolvimento para avaliação e verificação do cumprimento das normas da Resolução 466/12 e suas complementares.

Situação: Protocolo aprovado.

GOIANIA, 29 de Dezembro de 2014

Assinado por:

JOSE MARIO COELHO MORAES

(Coordenador)

Endereço: 1ª Avenida s/nº - Unidade de Pesquisa Clinica
Bairro: St. Leste Universitario **CEP:** 74.605-020
UF: GO **Município:** GOIANIA
Telefone: (62)3269-8338 **Fax:** (62)3269-8426 **E-mail:** cephcufg@yahoo.com.br
