# Supplementary material for: Association between severity of illicit drug dependence and quality of life in a psychosocial care center in BRAZIL: cross-sectional study
Source: Health Qual Life Outcomes. 2017 Nov 17;15:223. doi: 10.1186/s12955-017-0795-5 (PMC5693571; doi:10.1186/s12955-017-0795-5)
Supplement: Supplementary file 2 — Research consent by the institution “Psychosocial care center for alcohol and drugs” (CAPSad). (PDF 486 kb) [file 12955_2017_795_MOESM2_ESM.pdf]

Prefeitura
Municipal
de Goiânia

SECRETARIA MUNICIPAL DE SAÚDE

Goiânia, 13 de agosto de 2014

DECLARAÇÃO DE ANUÊNCIA

Declaro ter lido e concordar com o projeto de pesquisa **"QUALIDADE DE VIDA DOS USUÁRIOS DE ÁLCOOL E DROGAS ATENDIDOS NO CAPS AD CASA DE GOIÂNIA"** sob responsabilidade da pesquisadora **SELVA RIOS CAMPÊLO** e declaro conhecer e cumprir as Resoluções Éticas Brasileiras, em especial a Resolução CNS 466/2012. Esta instituição está ciente de suas responsabilidades como instituição coparticipante do presente projeto de pesquisa e de seu compromisso no resguardo da segurança e bem-estar de sujeitos de pesquisa nela recrutados, dispondo de infraestrutura necessária para a garantia de tal segurança e bem-estar. Estou ciente que a execução deste projeto dependerá da aprovação do mesmo pelo CEP da instituição proponente, mediante parecer ético consubstanciado e declaração de aprovação.

SECRETARIA MUNICIPAL DE SAÚDE
DGTES/ ESCOLA MUNICIPAL DE SAÚDE PÚBLI
Cristiane Oliveira C. de Albuquerque
Diretora
Decreto nº 793/2014

Cristiane Oliveira Cavalcante de Albuquerque
Secretaria Municipal de Saúde de Goiânia
Decreto 793/2014
